# Supplementary material for: Association of Periodontal Disease with the Occurrence of Unruptured Cerebral Aneurysm among Adults in Korea: A Nationwide Population-Based Cohort Study
Source: Medicina (Kaunas). 2021 Aug 30;57(9):910. doi: 10.3390/medicina57090910 (PMC8465273; doi:10.3390/medicina57090910)
Supplement: Supplementary file 1 [file medicina-57-00910-s001.zip › medicina-1345165-supplementary.pdf]

medicina

Method S1. Definition of comorbidity

Information on smoking status and alcohol intake were collected by questionnaire. Body mass index was defined as the participant's weight in kilograms divided by the square of the participant's height in meters. Regular physical activity was considered to be strenuous physical activity performed for at least 20 min more than once per week. Smoking status was categorized into none, former smoker and current smoker regardless of the amount of smoking. Socioeconomic status was dichotomized at the bottom 10%. Hypertension was defined using International Statistical Classification of Diseases Related Health Problems-10 (ICD-10) codes I10-I11 and the prescription of an antihypertensive agent with at least one claim per year. Diabetes mellitus was defined as main or secondary diagnosis of diabetes mellitus (ICD-10 codes E10-E14). Criteria for this diagnosis included at least one claim per year for both visiting an outpatient clinic and admission accompanied by prescription records for any hypoglycemic agents. Alternatively, at least one fasting plasma glucose ≥126 mg/dL. Dyslipidemia was defined using ICD-10 code E78 and the prescription of a lipid-lowering agent including statins with at least one claim per year. Kidney disease was identified by ICD-10 codes N18.1-N18.5 and N18.9 with at least one claim per year. Malignancy was defined by ICD-10 codes C00-D48 with at least one claim per year. Body mass index and blood and urine examination results were used with the baseline dataset [1].

Reference

1. Park SY, Kim SH, Kang SH, Yoon CH, Lee HJ, Yun PY, et al. Improved oral hygiene care attenuates the cardiovascular risk of oral health disease: a population-based study from Korea. Eur Heart J. 2019;40:1138-45.

Table S1. Multicollinearity assessment of the risk of unruptured cerebral aneurysm according to oral hygiene indicators.

Copyright: © 2021 by the authors. Licensee MDPI, Basel, Switzerland. This article is an open access article distributed under the terms and conditions of the Creative Commons Attribution (CC BY) license (http://creativecommons.org/licenses/by/4.0/).

	**Variance Inflation Factors**
Age (years)	1.129
Male sex	1.381
Socioeconomic status, % (*n*)	
Fifth quintile (highest)	-
Fourth quintile	1.224
Third quintile	1.231
Second quintile	1.217
First quintile (lowest)	1.228
Covered by medical aid	1.001
Regular physical activity	1.012
Alcohol intake	1.243
Smoking status	
Former smoker	1.104
Current smoker	1.237
Body mass index (kg/m^2)	1.087
Comorbidities	
Hypertension	1.864
Diabetes mellitus	1.275
Dyslipidemia	1.249
Renal disease	1.019
History of malignancy	1.018
Laboratory findings	
Total cholesterol	1.378

Fasting blood glucose level	1.273
Aspartate aminotransferase	1.142
Alanine aminotransferase	1.189
Gamma-glutamyl transferase	1.249
Proteinuria	1.014
Oral health status	
Presence of periodontal disease	1.118
Number of lost teeth	
0	-
1-5	1.044
≥6	1.124
Tooth brushing frequency per day	
0-1	-
2	2.013
≥3	2.198
Dental visits for any reason	1.487
Expert teeth scaling	1.514

Table S2. Comparison of demographic data between included and excluded participants.

Characteristics	Included participants	Excluded participants	*p* value	Standardized difference
Number of participants	209,620	305,246		
Age (years)	53.7 ± 8.7	53.8 ± 9.8	<.0001	0.051
Male sex	124,496 (59.4)	180,095 (59.0)	0.005	-0.097
Socioeconomic status			<.0001	0.124
Fifth quintile (highest)	83,828 (40.0)	93,100 (30.5)		
Fourth quintile	41,931 (20.0)	63,796 (20.9)		
Third quintile	29,697 (14.2)	50,976 (16.7)		
Second quintile	26,563 (12.7)	47,924 (15.7)		
First quintile (lowest)	27,171 (13.0)	49,145 (16.1)		
Covered by medical aid	430 (0.2)	350 (0.1)		

p value by Student's t-test and Chi-square test

Data are expressed as the mean ± standard deviation, or *n* (%).

Table S3. Baseline characteristics between participants with and without periodontal disease.

Characteristics	Periodontal disease		*p* value
	No	Yes	
Number of participants	165,814	43,806	
Age (years)	53.5 ± 8.8	54.3 ± 8.2	<.001
Male sex	95,102 (57.4)	29,394 (67.1)	<.001
Socioeconomic status			<.001
Fifth quintile (highest)	63,891 (38.5)	19,937 (45.5)	
Fourth quintile	33,319 (20.1)	8,612 (19.7)	
Third quintile	24,060 (14.5)	5,637 (12.9)	
Second quintile	21,855 (13.2)	4,708 (10.7)	
First quintile (lowest)	22,310 (13.5)	4,861 (11.1)	
Covered by medical aid	379 (0.2)	51 (0.1)	
Regular physical activity	16,476 (9.9)	4,779 (10.9)	<.001

Alcohol intake	71,754 (43.3)	20,907 (47.7)	<.001
Body mass index (kg/m^2)	23.9 ± 2.9	24.1 ± 2.8	<.001
Systolic blood pressure (mmHg)	125.8 ± 16.8	125.8 ± 16.0	0.876
Diastolic blood pressure (mmHg)	78.6 ± 10.8	78.6 ± 10.5	0.522
Comorbidities			
Hypertension	60,610 (36.6)	17,007 (38.8)	<.001
Diabetes mellitus	15,575 (9.4)	5,725 (13.1)	<.001
Dyslipidemia	29,483 (17.8)	8,890 (20.3)	<.001
Current smoker	33,726 (20.3)	10,695 (24.4)	<.001
Renal disease	436 (0.3)	150 (0.3)	0.005
History of malignancy	18,768 (11.3)	6,577 (15.0)	<.001
Laboratory findings			
Total cholesterol (mg/dL)	197.8 ± 36.3	197.7 ± 36.2	0.557
Fasting blood glucose level (mg/dL)	97.3 ± 26.9	99.7 ± 29.0	<.001
Aspartate aminotransferase (U/L)	26.5 ± 16.2	26.6 ± 15.5	0.299
Alanine aminotransferase (U/L)	25.4 ± 20.0	26.2 ± 20.3	<.001
Gamma-glutamyl transferase (U/L)	38.1 ± 55.3	40.0 ± 51.4	<.001
Proteinuria (≥1+ in dip stick test)	5,315 (3.2)	1,524 (3.5)	0.004
Oral hygiene parameters			
Number of lost teeth			<.001
0	127,105 (76.7)	31,008 (70.8)	
1-5	32,208 (19.4)	11,083 (25.3)	
≥6	6,501 (3.9)	1,715 (3.9)	
Tooth brushing frequency per day			<.001
0-1	24,312 (14.7)	5,571 (12.7)	
2	74,067 (44.7)	17,559 (40.1)	
≥3	67,435 (40.7)	20,676 (47.2)	
Dental visits for any reason	63,675 (38.4)	28,965 (66.1)	<.001
Expert teeth scaling	35,711 (21.5)	18,544 (42.3)	<.001

Note: p value by Student's t-test and Chi-square test. Data are expressed as the mean ± standard deviation, or *n* (%).

Table S4. The subgroup analysis regarding periodontal disease and occurrence of unruptured cerebral aneurysm according to age and sex.

	**Model 1**		**Model 2**		**Model 3**	
	HR (95% CI)	*p* value for interaction effect	HR (95% CI)	*p* value for interaction effect	HR (95% CI)	*p* value for interaction effect
Age		0.542		0.678		0.798
≤51	1.12 (1.07-1.18)		1.14 (1.10-1.21)		1.14 (1.07-1.24)	
≥52	1.14 (0.99-1.06)		1.15 (1.04-1.24)		1.16 (1.08-1.23)	
Sex		0.872		0.932		0.913
Male	1.05 (1.03-1.10)		1.08 (1.05-1.13)		1.09 (1.06-1.15)	
Female	1.10 (1.02-1.14)		1.14 (1.06-1.21)		1.13 (1.06-1.16)	

The multivariate model (1) was undertaken for the association of each oral hygiene parameter with the occurrence of unruptured cerebral aneurysm adjusted for socioeconomic status, regular physical activity, alcohol intake, smoking status, body mass index (kg/m^2), hypertension, diabetes mellitus, dyslipidemia, renal disease, and history of malignancy

The multivariate model (2) was undertaken for the association of each oral hygiene parameter with the occurrence of unruptured cerebral aneurysm adjusted for the variables in model 1 as well as systolic blood pressure, total cholesterol, fasting blood glucose level, aspartate aminotransferase, alanine aminotransferase, gamma-glutamyl transferase, and proteinuria

The multivariate model (3) was undertaken for the association of each oral hygiene parameter with the occurrence of unruptured cerebral aneurysm adjusted for the variables in model 2 as well as overall oral hygiene indicators (presence of periodontal disease, number of lost teeth, tooth brushing frequency per day, dental visits for any reason, and expert teeth scaling).

HR, hazard ratio; CI, confidence interval.

Table S5. The subgroup analysis regarding periodontal disease and occurrence of unruptured cerebral aneurysm according to smoking status.

	**Model 1**		**Model 2**		**Model 3**	
	HR (95% CI)	*p* value for interaction effect	HR (95% CI)	*p* value for interaction effect	HR (95% CI)	*p* value for interaction effect
Smoking status		0.768		0.659		0.713
Former smoker	1.14 (1.05-1.25)		1.11 (1.03-1.24)		1.12 (1.05-1.21)	
Current smoker	1.25 (1.02-1.40)		1.23 (1.05-1.44)		1.22 (1.06-1.38)	

The multivariate model (1) was undertaken for the association of each oral hygiene parameter with the occurrence of unruptured cerebral aneurysm adjusted for socioeconomic status, regular physical activity, alcohol intake, smoking status, body mass index (kg/m^2), hypertension, diabetes mellitus, dyslipidemia, renal disease, and history of malignancy

The multivariate model (2) was undertaken for the association of each oral hygiene parameter with the occurrence of unruptured cerebral aneurysm adjusted for the variables in model 1 as well as systolic blood pressure, total cholesterol, fasting blood glucose level, aspartate aminotransferase, alanine aminotransferase, gamma-glutamyl transferase, and proteinuria

The multivariate model (3) was undertaken for the association of each oral hygiene parameter with the occurrence of unruptured cerebral aneurysm adjusted for the variables in model 2 as well as overall oral hygiene indicators (presence of periodontal disease, number of lost teeth, tooth brushing frequency per day, dental visits for any reason, and expert teeth scaling).

HR, hazard ratio; CI, confidence interval
